# BK channels regulate extracellular Tat-mediated HIV-1 LTR transactivation

**DOI:** 10.1038/s41598-019-48777-y

**Published:** 2019-08-22

**Authors:** Nabab Khan, Koffi L. Lakpa, Peter W. Halcrow, Zahra Afghah, Nicole M. Miller, Jonathan D. Geiger, Xuesong Chen

**Affiliations:** Department of Biomedical Sciences, University of North Dakota School of Medicine and Health Sciences, Grand Forks, ND 58203 USA

**Keywords:** Retrovirus, Lysosomes

## Abstract

HIV-1 Tat is essential for HIV-1 replication and plays an important role in latent HIV-1 infection, HIV-1 associated neurological complication, and other HIV-1 comorbidities. Secreted from HIV-1 infected or transfected cells, Tat can be up-taken into cells by receptor-mediated endocytosis and internalized into endolysosomes. To reach nucleus where it can facilitate HIV-1 viral replication, exogenous Tat has to escape the degradation by endolysosomes. Because of findings that endolysosome de-acidification with, for example, the weak-base anti-malarial drug chloroquine prevents exogenous Tat degradation and enhances the amount of Tat available to activate HIV-1 LTR, we hypothesize that acidifying endolysosomes may enhance Tat degradation in endolysosomes and restrict LTR transactivation. Here, we determined the involvement of endolysosome-resident transient receptor potential mucolipin 1 channel (TRPML1) and the big conductance Ca^2+^-activated potassium (BK) channel in regulating endolysosome pH, as well as Tat-mediated HIV-1 LTR transactivation in U87MG cells stably integrated with HIV-1 LTR luciferase reporter. Activating TRPML1 channels with ML-SA1 acidified endolysosomes and restricted Tat-mediated HIV-1 LTR transactivation. These effects of ML-SA1 appeared to be mediated through activation of BK channels, because the effects of ML-SA1 on Tat-mediated HIV-1 LTR transactivation were blocked using pharmacological inhibitors or shRNA knock-down of BK channels. On the other hand, activating TRPML1 and BK channels enhanced cellular degradation of exogenous Tat. These results suggest that acidifying endolysosomes by activating TRPML1 or BK channels may provide therapeutic benefit against latent HIV-1 infection, HIV-1 associated neurocognitive disorders, and other HIV-1 comorbidities.

## Introduction

Infecting 37 million people globally^1^, HIV-1 enters the CNS within weeks of infection^2,3^ and can harbor in CSF, perivascular macrophages, microglia, and astrocytes^4^. Although combined antiretroviral therapy (ART) effectively suppresses HIV-1 replication, it does not completely eliminate the virus. In this ART era, reservoirs of HIV-1 exist centrally and peripherally^5,6^, low levels of neuroinflammation persist, and the prevalence of HIV associated neurocognitive disorders (HAND) remains high (30–50%)^7,8^. The existence of viral reservoirs makes complete eradication of HIV-1 extremely challenging^9–11^. Therefore, additional strategies are needed to block viral reactivation in sanctuary sites and to prevent disease progression including HAND. Because ART does not block Tat secretion from HIV-1 infected cells^12^, and brain levels of Tat remain elevated even when HIV-1 levels are below detectable levels with ART^13^, one strategy might be to prevent Tat from activating HIV-1 replication through elongation of the HIV-1 long terminal repeat (LTR)^14–16^.

Two-thirds of cellular Tat can be secreted from HIV-1 infected or transfected cells^17–20^ and extracellular Tat crosses plasma membranes by various mechanisms including endocytosis; a major pathway for Tat entry^21,22^ following interactions with specific cell surface proteins and receptors^21–26^. Once internalized into endolysosomes, Tat has to escape from endolysosomes into the cytosol before it transits to the nucleus and activates the HIV-1 LTR promoter^27–29^. Typically, robust HIV-1 LTR transactivation requires high concentrations of exogenous Tat and disruption of plasma membranes using, for example, scrape-loading methods^22,28,30^. When endogenously expressed in cytosol, Tat can be imported directly into nucleus and activate HIV-1 LTR transactivation using importin β-dependent nuclear localization signals^31^. In contrast, secreted Tat or exogenously added Tat has to first enter the endolysosome system via endocytosis. Thus, avoiding endolysosome degradation is critical for exogenous Tat to first escape endolysosomes and then enter nucleus to activate HIV-1 Tat LTR transactivation. Consistent with findings of others^22,29,30,32,33^, we found that the lysosomotropic agent chloroquine enhanced extracellular Tat-mediated HIV-1 LTR transactivation^34^. Given that chloroquine does not increase^22^, or even decrease^35^, HIV-1 LTR transactivation under conditions when Tat is expressed intracellularly, it is generally thought that chloroquine, a weak base, neutralizes the acidic pH of endolysosomes and prevents exogenous HIV-1 Tat degradation, thus increasing the amount of Tat available to activate HIV-1 LTR in nucleus.

Thus, acidifying endolysosomes could enhance HIV-1 Tat degradation in endolysosomes, preventing Tat escape from endolysosomes and blocking subsequent activation of HIV-1 LTR in the nucleus. The acidic endolysosome luminal pH is maintained by the electrogenic pumping of protons by vacuolar-ATPase (v-ATPase) in conjunction with chloride and other ions^36,37^. Others and we have found that activating endolysosome-resident transient receptor potential mucolipin 1 channel (TRPML1) channels with the agonist ML-SA1 resulted in endolysosome acidification^38,39^. In the present studies, we determined the involvement of TRPML1 and the big conductance Ca^2+^-activated potassium (BK) channel in regulating endolysosome pH, as well as extracellular Tat-mediated HIV-1 LTR transactivation and cellular degradation of extracellularly added Tat. TRPML1 and BK channel activation acidified endolysosomes, enhanced cellular degradation of extracellularly added Tat, and restricted Tat-mediated HIV-1 LTR transactivation. Thus, TRPML1 and BK channels might be targeted therapeutically to prevent re-activation of latent HIV-1 infection, and to decrease the prevalence and severity of HIV-1 associated neurocognitive disorders and other HIV-1 comorbidities.

## Results

### Activating TRPML1 with ML-SA1 acidifies endolysosomes and restricts Tat-mediated HIV-1 LTR transactivation

**Figure 1:**
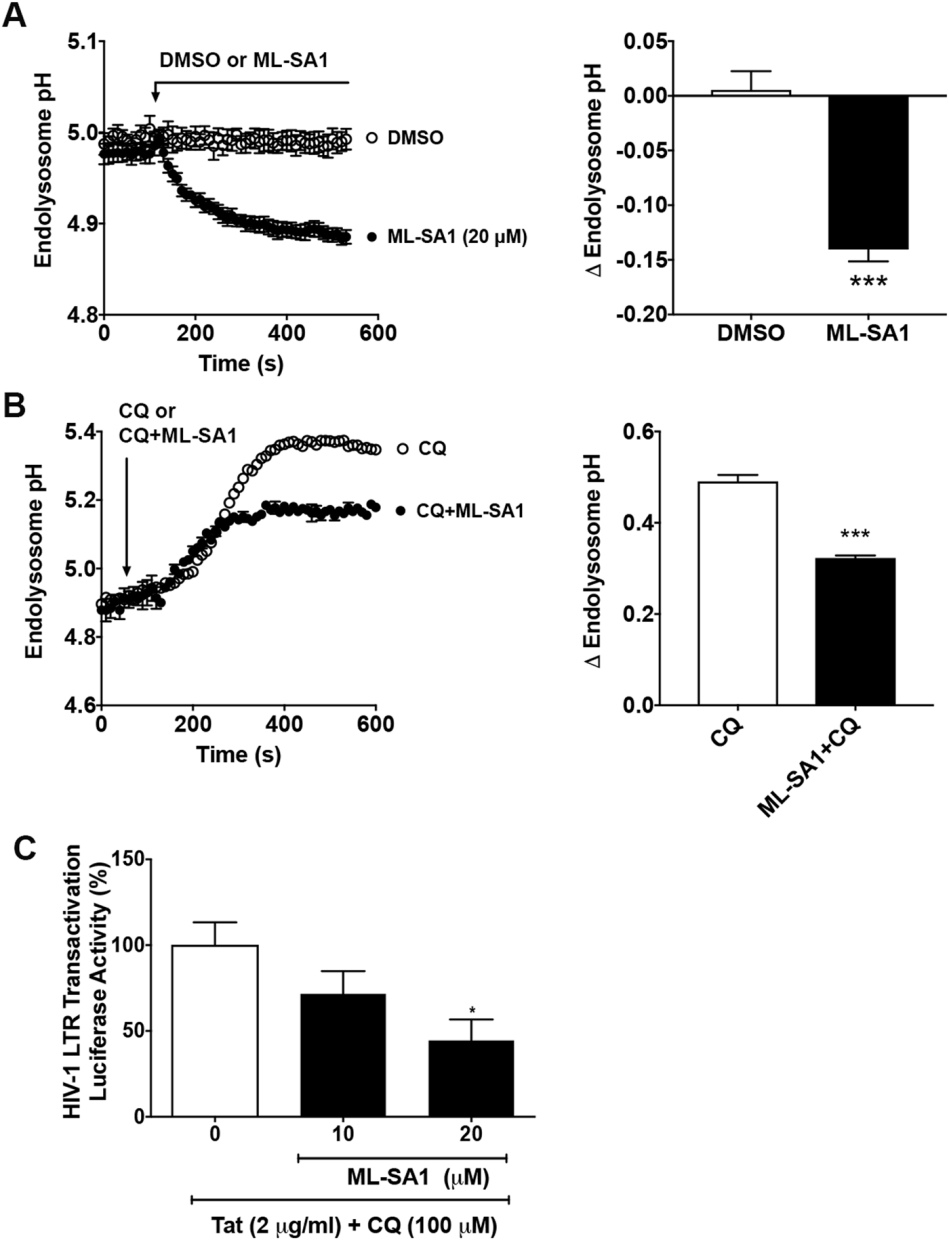
Activating TRPML1 with ML-SA1 acidified endolysosomes and restricted Tat-mediated LTR transactivation. (**A**) Endolysosome pH was measured ratiometrically with LysoSensor. As shown in the representative endolysosome pH tracing and bar graph, ML-SA1 (20 μM) significantly decreased endolysosome pH in U87MG cells, when compared with DMSO control (n = 12 cells from 2 experimental replicates, ***p < 0.001). (**B**) As shown in the representative endolysosome pH tracing and bar graph, co-treatment of ML-SA1 (20 μM) with chloroquine (CQ, 100 μM) decreased endolysosome pH in U87MG cells, when compared with chloroquine (CQ, 100 μM) treatment alone (n = 23 cells from 3 experimental replicates, ***p < 0.001). (**C**) ML-SA1 treatment (10 and 20 μM for 48 hr) restricted Tat (2 μg/ml)-mediated LTR transactivation in the presence of CQ in U87MG cells stably transfected with an integrated luciferase gene under the control of an HIV-1 LTR promoter (n = 3 experimental replicates, *p < 0.05).

Endolysosome pH is largely maintained by v-ATPases that pump protons into endolysosomes; v-ATPase activity is regulated by ions including chloride and calcium^36,37^. Recent studies have shown that ML-SA1 activation of TRPML1, a cation channel residing on endolysosomes, decreases endolysosome pH^38–40^. Accordingly, it was important for us to first confirm using U87MG cells the extent to which ML-SA1 affects endolysosome pH. The concentration of 20 μM was used because at this concentration ML-SA1 protects against lysosomal lipid accumulation in Niemann-Pick type C^40^ and clears intraneuronal accumulation of amyloid beta^38,39^. We found that ML-SA1 (20 μM) decreased significantly (p < 0.001) endolysosome pH by about 0.15 pH units (Fig. 1A); a decrease in pH that represents a 20–30% increase in proton concentration. ML-SA1 (20 μM) also blunted significantly (p < 0.001) chloroquine-induced increases in endolysosome pH (Fig. 1B). And finally, ML-SA1 (20 μM) restricted significantly (p < 0.05) Tat-mediated HIV-1 LTR transactivation in the presence of 100 μM of chloroquine (Fig. 1C).

### TRPML-1 knockdown prevents ML-SA1’s effects on Tat mediated HIV-1 LTR transactivation

**Figure 2:**
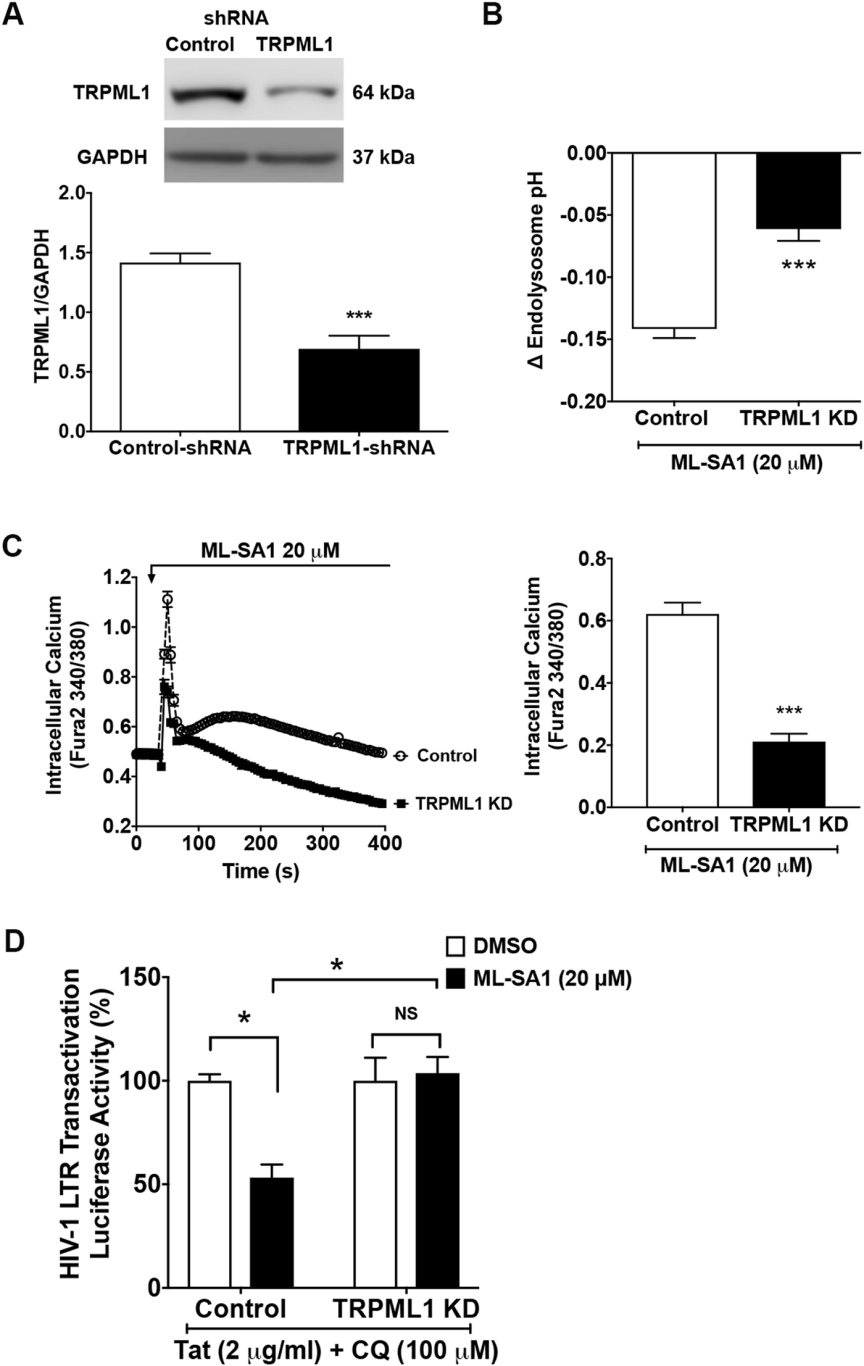
TRPML1 knockdown blocked ML-SA1’s restriction of Tat-mediated LTR transactivation. (**A**) As shown in the representative immunoblots (GAPDH as loading control) and bar graph, U87MG cells stably transfected with TRPML1-shRNA exhibited decreased protein levels of TRPML1, when compared with cells stably transfected with control-shRNA (n = 3 experimental replicates, ***p < 0.001). (**B**) The degree of ML-SA1 (20 μM)-induced decreases in endolysosome pH was reduced in TRPML1 knockdown cells, when compared with cells stably transfected with control-shRNA (n = 27 cells from 3 experimental replicates, ***p < 0.001). (**C**) Intracellular calcium was measured with Fura-2 under calcium free condition. As shown in the representative calcium tracing and bar graph, the degree of ML-SA1 (20 μM)-induced increases in intracellular calcium levels was reduced in TRPML1 knockdown cells, when compared with cells stably transfected with control-shRNA (n = 16 cells from 2 experimental replicates, ***p < 0.001). (**D**) Compared with DMSO control, ML-SA1 (20 μM for 48 hr) restricted Tat-mediated LTR transactivation (in the presence of chloroquine) in U87MG cells stably transfected with control-shRNA. However, ML-SA1 (20 μM) failed to restrict Tat-mediated LTR transactivation (in the presence of chloroquine) in TRPML1 knockdown cells (n = 3 experimental replicates, *p < 0.05, ^NS^p > 0.05).

We next determined the specificity of ML-SA1’s actions by determining the effects of TRPML1 knockdown on Tat-mediated HIV-1 LTR transactivation. Using shRNA and selection pressure methods, we generated stable TRPML1 knockdown cells. As confirmed by immunoblotting, TRPML1 shRNA significantly (p < 0.001) decreased protein levels of TRPML1 (Fig. 2A and Supplementary Fig. S1). We next determined the extent to which TRPML1 knockdown affected endolysosome pH; TRPML1 knockdown by itself did not affect baseline endolysosome pH (data not shown) but did significantly (p < 0.001) attenuate ML-SA1-induced decreases in endolysosome pH (Fig. 2B). We also demonstrated that TRPML1 knockdown decreased significantly (p < 0.001) ML-SA1-induced increases in intracellular free calcium under calcium-free conditions (Fig. 2C). And, finally, we found that TRPML1 knockdown by itself did not significantly affect Tat-mediated LTR transactivation but did significantly (p < 0.05) block ML-SA1-induced reductions in Tat-mediated LTR transactivation (Fig. 2D).

### BK channel involvement in the action of ML-SA1 in restricting Tat-mediated HIV-1 LTR transactivation

**Figure 3:**
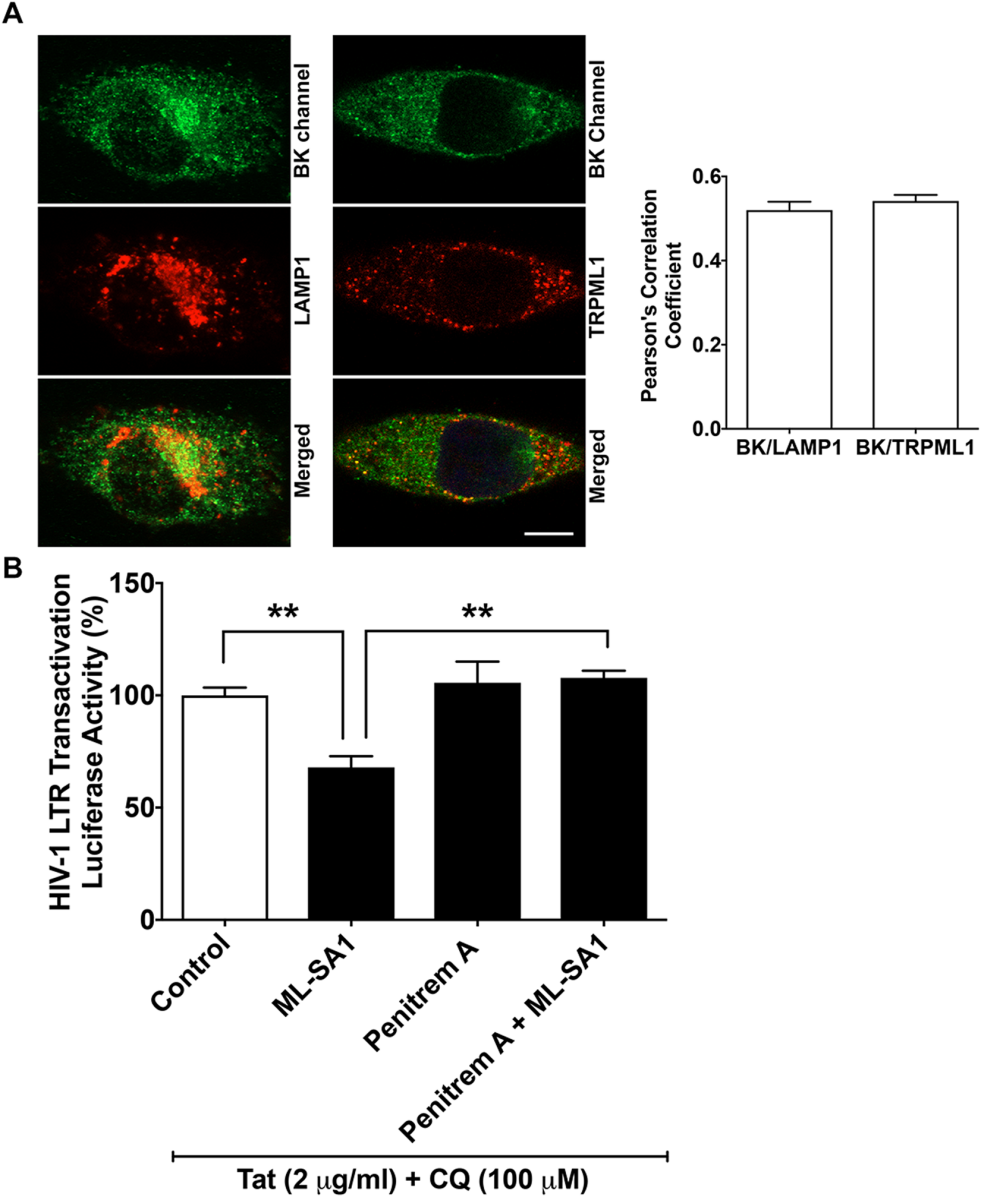
BK channel antagonist prevented ML-SA1 restriction of Tat-mediated LTR transactivation. (**A**) As shown in the representative double-staining confocal images, BK channels colocalized with LAMP1 with a Pearson’s correlation coefficient of 0.54 (n = 6 cells from 2 experimental replicates, bar = 10 μm). BK channels also colocalized with TRPML1 with a Pearson’s correlation coefficient of 0.52 (n = 6 cells from 2 experimental replicates, bar = 10 μm). (**B**) BK channel antagonist penitrem A treatment (1 μM for 48 h) alone did not affect Tat-mediated LTR transactivation in the presence of chloroquine, when compared with DMSO control (n = 3 experimental replicates, p > 0.05). When compared with ML-SA1 (20 μM) treatment alone, co-treatment of ML-SA1 (20 μM) with penitrem A (1 μM) failed to restrict Tat-mediated LTR transactivation in the presence of chloroquine in U87MG cells (n = 3 experimental replicates, **p < 0.01).

**Figure 4:**
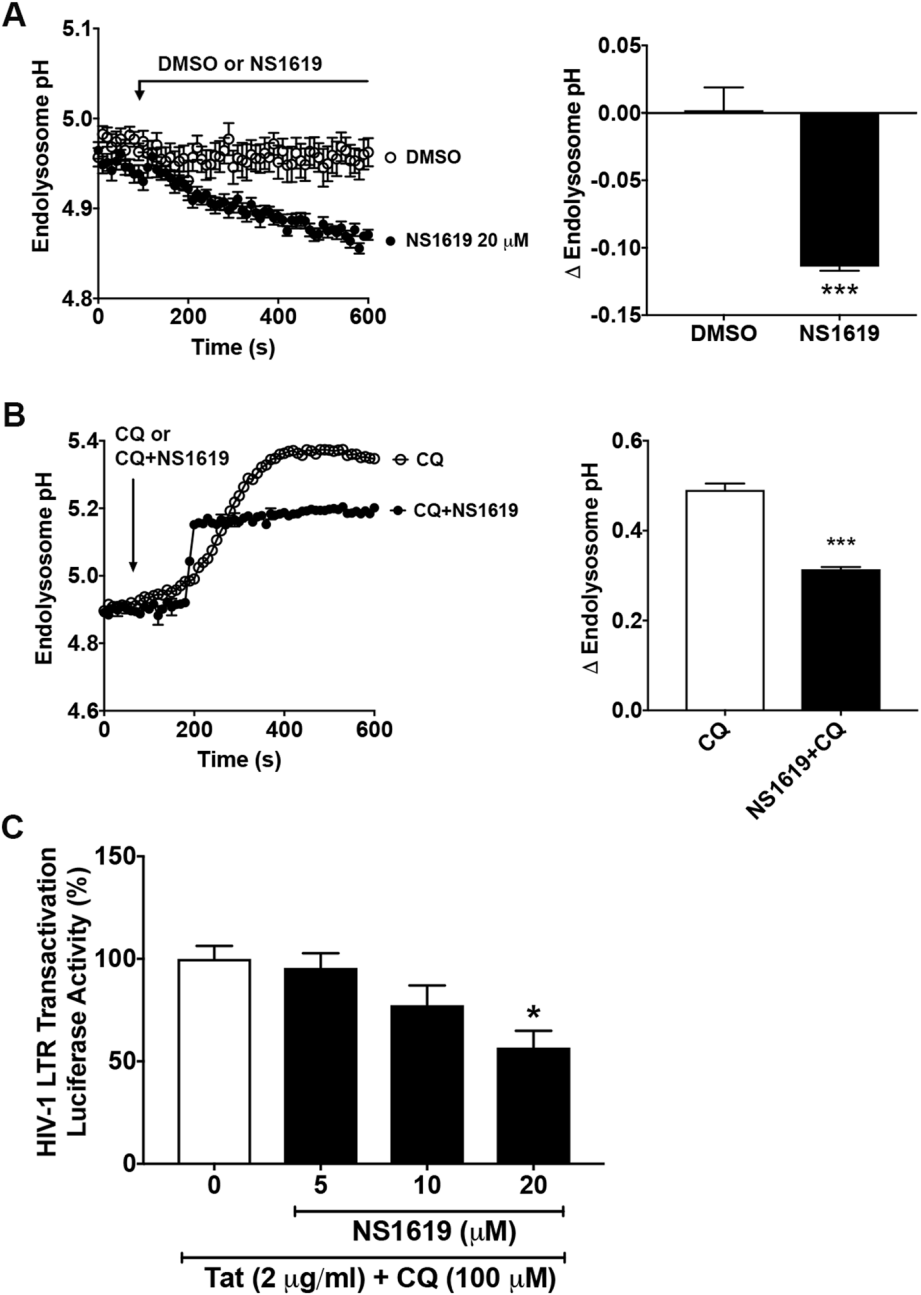
BK channel activator NS1619 acidified endolysosomes and restricted Tat-mediated LTR transactivation. (**A**) Endolysosome pH was measured ratiometrically with LysoSensor. As shown in the representative endolysosome pH tracing and bar graph, NS1619 (20 μM), a BK channel activator, significantly decreased endolysosome pH in U87MG cells, when compared with DMSO control (n = 18 cells from 3 experimental replicates, ***p < 0.001). (**B**) As shown in the representative endolysosome pH tracing and bar graph, co-treatment of NS1619 (20 μM) with chloroquine (CQ, 100 μM) decreased endolysosome pH in U87MG cells, when compared to chloroquine (CQ, 100 μM) treatment alone (n = 12 cells from 2 experimental replicates, ***p < 0.001). (**C**) NS1619 treatment (5, 10, and 20 μM for 48 h) restricted Tat (2 μg/ml)-mediated LTR transactivation in the presence of CQ in U87MG cells stably transfected with an integrated luciferase gene under the control of an HIV-1 LTR promoter (n = 3 experimental replicates, *p < 0.05).

Next, we explored underlying mechanisms whereby activating TRPML1 restricts Tat-mediated LTR transactivation. Because calcium release through TRPML1 channels activates BK channels^41,42^, we determined next the involvement of BK channels in ML-SA1-induced acidification of endolysosomes and reduction in Tat-mediated LTR transactivation. Using immunostaining methods, we demonstrated that BK channels co-localized with LAMP1 and TRPML-1 with similar Pearson’s correlation coefficients (Fig. 3A). Using a pharmacological approach, we then determined the extent to which blocking BK channels affected ML-SA1’s effects on Tat-mediated LTR transactivation. Blocking BK channels with penitrem A (1 μM) reversed significantly (p < 0.01) the restrictive effects of ML-SA1 (p < 0.01) on Tat-mediated LTR transactivation (Fig. 3B). To further determine the involvement of BK channels we used a selective activator of BK channels, NS1619, and found that NS1619 (20 μM) significantly (p < 0.001) acidified endolysosome pH (Fig. 4A) and attenuated significantly (p < 0.001) chloroquine-induced increases in endolysosome pH (Fig. 4B). And finally, NS1619 decreased significantly (p < 0.05) Tat-mediated HIV-1 LTR transactivation in the presence of 100 μM of chloroquine (Fig. 4C). Together, these findings indicate that BK channels are involved in ML-SA1-induced acidification of endolysosomes and reduction in Tat-mediated LTR transactivation.

### BK channel knockdown prevents ML-SA1- and NS1619-induced reduction in Tat-mediated HIV-1 LTR transactivation

**Figure 5:**
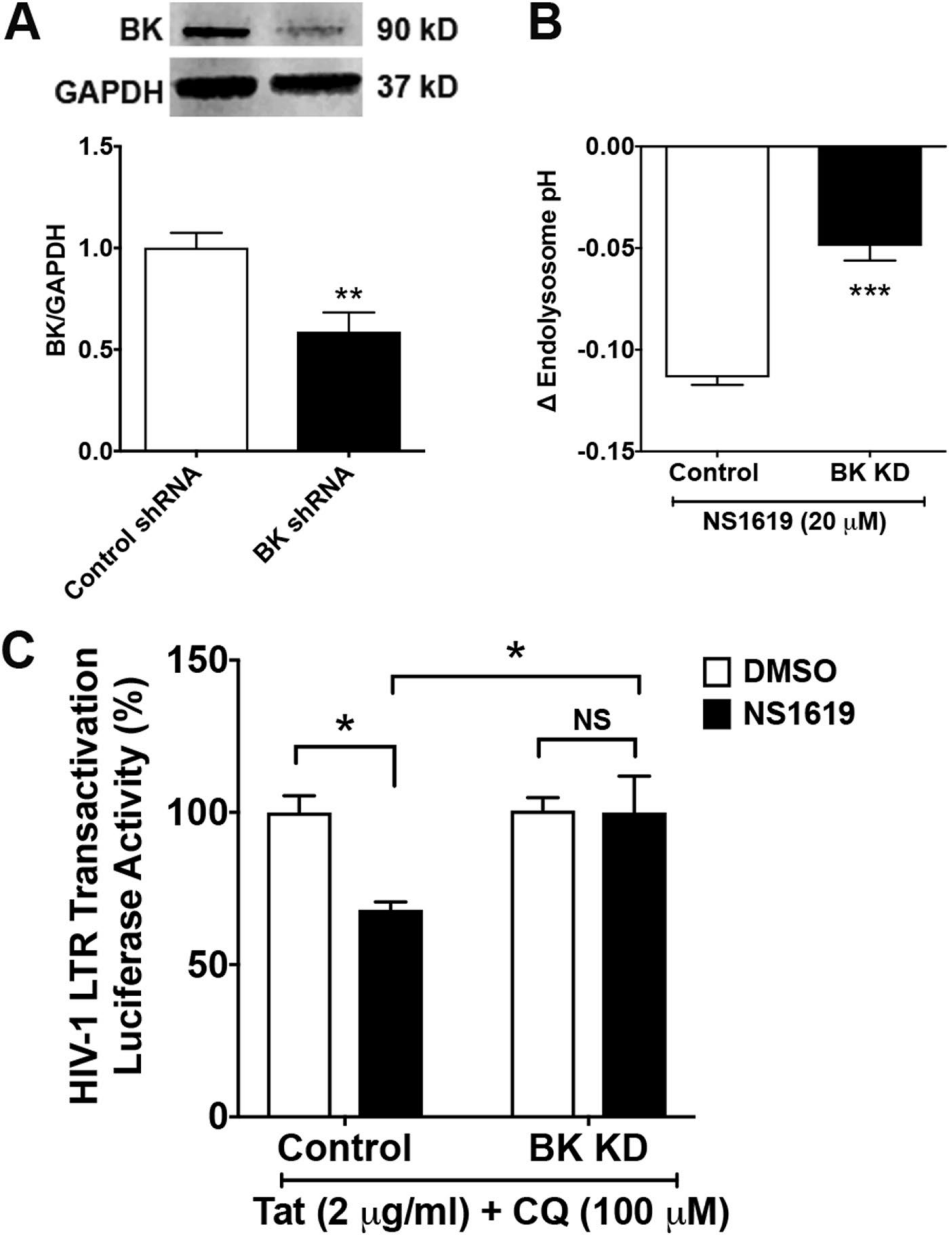
BK channel knockdown blocks NS1619’s effect in restricting Tat-mediated LTR transactivation. (**A**) As shown in the representative immunoblots (GAPDH as loading control) and bar graph, U87MG cells stably transfected with BK channel shRNA exhibited decreased protein levels of BK channel, when compared with cells stably transfected with control-shRNA (n = 4 experimental replicates, **p < 0.01). (**B**) The degree of NS1619 (20 μM)-induced decreases in endolysosome pH was reduced in BK channel knockdown cells, when compared with cells stably transfected with control-shRNA (n = 21 cells from 3 experimental replicates, ***p < 0.001). (**C**) Compared with DMSO control, NS1619 (20 μM for 48 h) restricted Tat-mediated LTR transactivation (in the presence of chloroquine) in U87MG cells stably transfected with control-shRNA. However, NS1619 (20 μM) failed to restrict Tat-mediated LTR transactivation (in the presence of chloroquine) in BK channel knockdown cells (n = 3 experimental replicates, *p < 0.05, ^NS^p > 0.05).

We next determined the extent to which BK channel knockdown affects Tat-mediated LTR transactivation. Using shRNA, we generated stable BK channel knockdown cells, as confirmed by significantly (p < 0.01) decreased protein expression levels of BK channels (Fig. 5A and Supplementary Fig. S2). Functionally, BK channel knockdown decreased significantly (p < 0.001) the ability of NS1619 to acidify endolysosomes (Fig. 5B). NS1619 (20 μM) also failed to restrict Tat-mediated LTR transactivation in BK channel knockdown cells (Fig. 5C).

**Figure 6:**
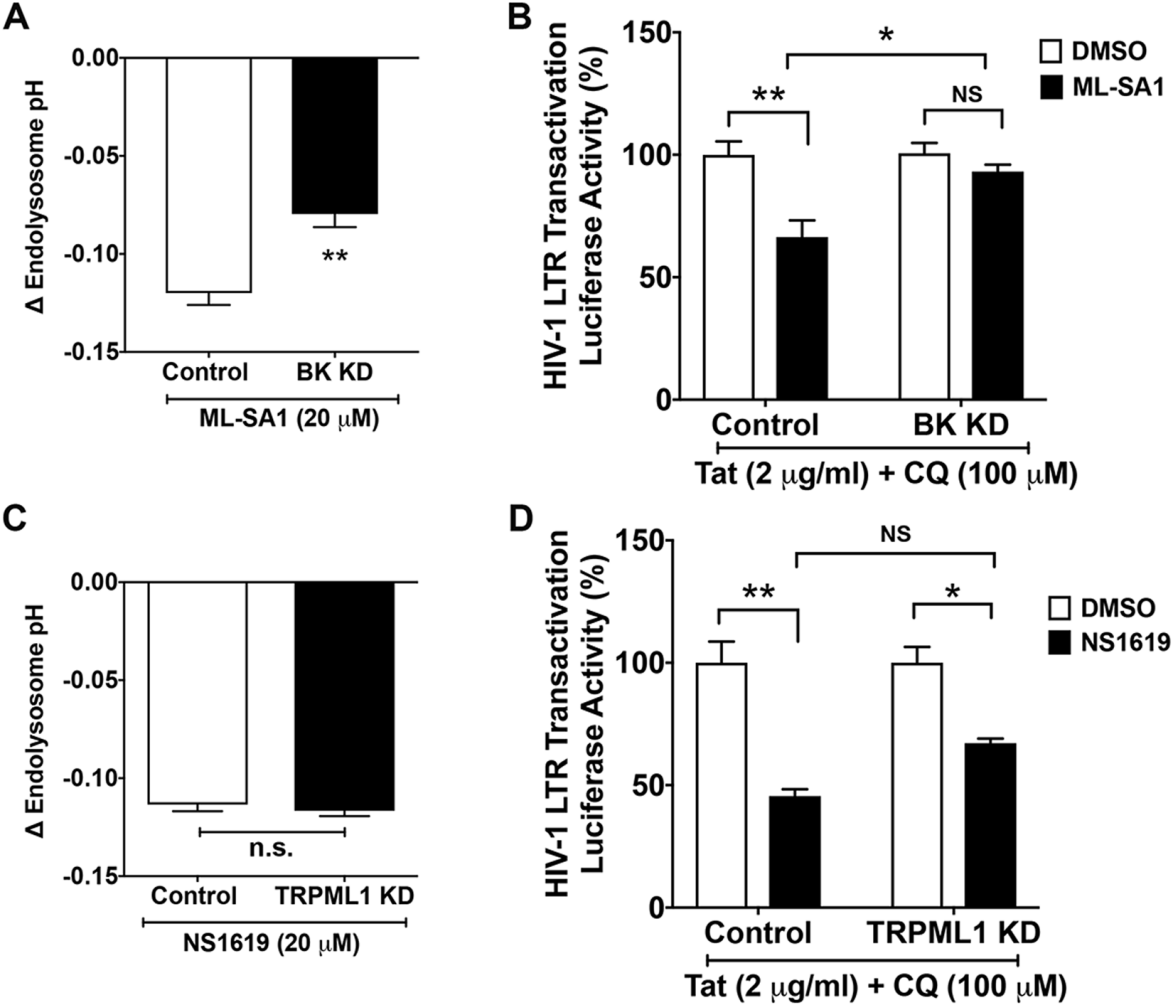
ML-SA1 restriction of Tat-mediated LTR transactivation is dependent on activation of BK channels. (**A**) The degree of ML-SA1 (20 μM)-induced decreases in endolysosome pH was reduced in BK channel knockdown cells, when compared with that of U87MG cells stably transfected with control-shRNA (n = 15 cells from 2 experimental replicates, **p < 0.01). (**B**) Compared with DMSO control, ML-SA1 (20 μM for 48 h) restricted Tat-mediated LTR transactivation (in the presence of chloroquine) in U87MG cells stably transfected with control-shRNA (n = 3 experimental replicates, **p < 0.01). However, ML-SA1 (20 μM) failed to restrict Tat-mediated LTR transactivation (in the presence of chloroquine) in BK channel knockdown cells (n = 3 experimental replicates, *p < 0.05, ^NS^p > 0.05). (**C**) The degree of NS1619 (20 μM)-induced decreases in endolysosome pH was similar in TRPML1 knockdown cells as that of U87MG cells stably transfected with control-shRNA (n = 17 cells from 2 experimental replicates, ^ns^p > 0.05). (**D**) Compared with DMSO control, NS1619 (20 μM for 48 h) restricted Tat-mediated LTR transactivation (in the presence of chloroquine) in U87MG cells stably transfected with control-shRNA (n = 3 experimental replicates, **p < 0.01). NS1619 (20 μM) was still able to restrict Tat-mediated LTR transactivation (in the presence of chloroquine) in TRPML1 knockdown cells (n = 3 experimental replicates, *p < 0.05, ^NS^p > 0.05).

Next, we determined potential functional interactions between TRPML1 and BK channels. BK channel knockdown decreased significantly (p < 0.01) the ability of ML-SA1 to acidify endolysosomes (Fig. 6A) and prevented significantly (p < 0.05) the restrictive effect of ML-SA1 on Tat-mediated LTR transactivation (Fig. 6B). On the other hand, using TRPML1 knockdown cells, we demonstrated that TRPML1 knockdown did not affect endolysosome acidifying effects of NS1619 (Fig. 6C), nor did TRPML1 knockdown affect NS1619-induced restriction of Tat-mediated LTR transactivation (Fig. 6D). These findings indicate that TRPML1 activator-induced endolysosome acidification and restriction in Tat-mediated LTR transactivation is dependent on activation of BK channels.

### ML-SA1and NS1619 enhance cellular degradation of exogenous Tat

**Figure 7:**
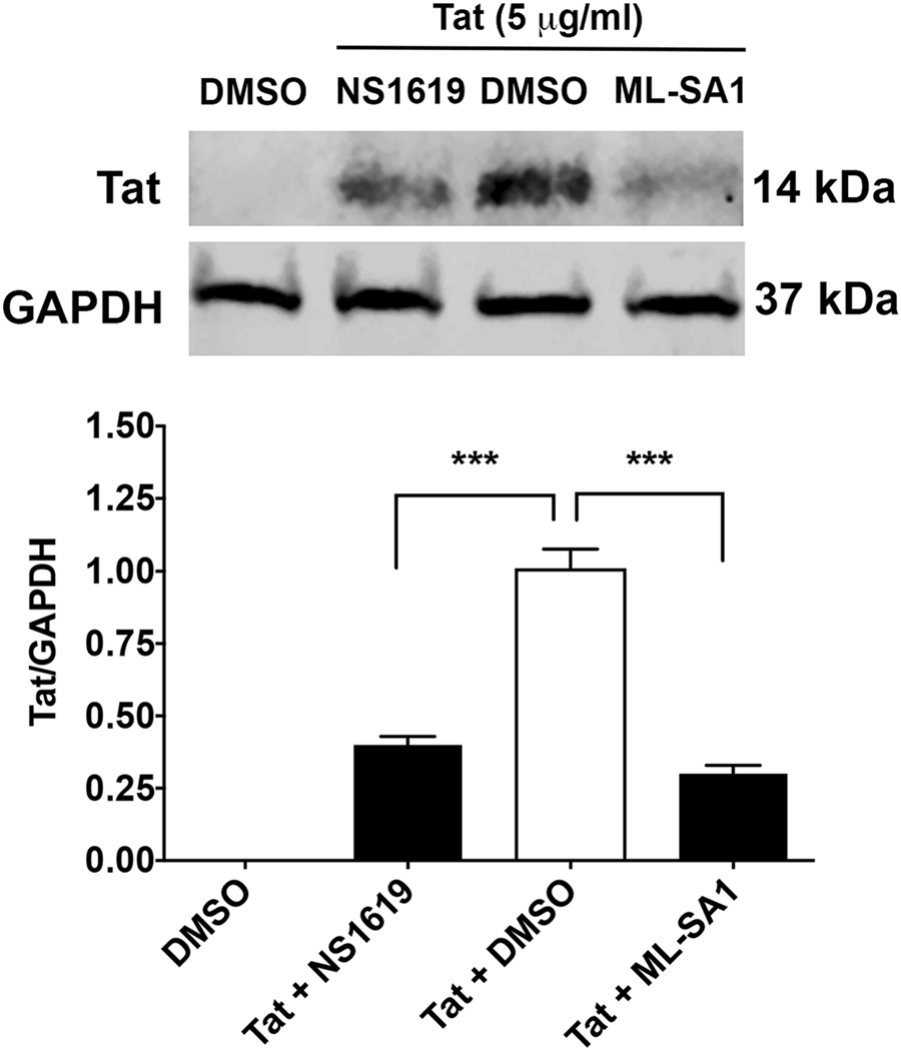
ML-SA1 and NS1619 enhanced cellular degradation of extracellularly added Tat. U87MG cells were treated with DMSO, NS1619 (20 μM), or ML-SA1(20 μM) in the presence of high levels of Tat (5.0 μg/ml) and chloroquine (100 μM) for 24 h. Quantitative immunoblotting showed that co-treatment of Tat with NS1619 or ML-SA1 significantly decreased cellular levels of exogenous Tat, when compared with co-treatment of Tat with DMSO (n = 3 experimental replicates, ***p < 0.001).

Chloroquine neutralizes the acidic pH of endolysosomes and thereby may prevent exogenous Tat degradation and increase the amount of Tat available to activate HIV-1 LTR in nucleus^22,29,30,32,33^. As such, acidifying endolysosomes with ML-SA1 and NS1619 could enhance cellular degradation of exogenously added Tat and decrease the amount of Tat available to activate HIV-1 LTR in nucleus. Here, we tested this hypothesis by directly measuring cellular protein levels of Tat using immunoblotting methods. Cells were co-treated with 5.0 μg/ml of Tat and DMSO as a control, ML-SA1 (20 μM), or NS1619 (20 μM) for 24 h in the presence of chloroquine (100 μM). ML-SA1 and NS1619 significantly (p < 0.001) decreased cellular protein levels of Tat (Fig. 7 and Supplementary Fig. S3), and no detectable levels of Tat protein in media were found for any of the treatments. Together, our findings suggest that acidifying endolysosome with ML-SA1 and NS1619 could enhance cellular degradation of exogenous added Tat and decrease cellular levels of Tat available to activate HIV-1 LTR in the nucleus.

## Discussion

Extracellular macromolecules and plasma membrane components can enter cells by endocytosis where they can be targeted for degradation by trafficking through endosomes into lysosomes^37,43–45^. Increasingly appreciated is the complex and dynamic nature of the endolysosome system; it is morphologically diverse, physiologically important, pathologically relevant, and participates in complex inter-organellar signaling^46–49^. An important hallmark of endolysosomes is their acidic luminal pH, which is maintained by the v-ATPase that pumps proton into the lumen of endolysosomes and the influx of chloride into the lumen^36,37^. Others and we have shown that endolysosome de-acidification prevented endolysosome degradation of exogenously administered HIV-1 Tat and that this resulted in increases in the amount of Tat available to activate HIV-1 LTR^22,29,30,32–34^. However, no one has reported the extent to which and the mechanisms by which endolysosome acidification enhances Tat degradation and decreases the amount of Tat to activate HIV-1 LTR. Here, we demonstrated that activation of TRPML1 and BK channels acidified endolysosomes, enhanced cellular degradation of exogenously administered Tat, and decreased the activation of HIV-1 LTR.

The acidic environment of endolysosome is not only important for the proper function of endolysosome-resident acidic hydrolases, but also important for other physiological functions such vesicular trafficking, the proper processing of antigens, and the sensing of cellular nutrients^50–52^. The v-ATPase is one of the major regulators of endolysosome pH and its activity is affected by its expression levels, its isoforms, and associations between its V0 and V1 components^53^. Endolysosome pH is also affected by proton leak mechanisms^54^. Many factors can de-acidify endolysosome pH. Weak-bases, such as NH_4_Cl, chloroquine, and methylamine, can accumulate in acidic environment of endolysosomes and neutralize the endolysosome pH^55,56^. Ionophores like monensin and nigericin can exchange other ions with proton, leading to leakage of protons from endolysosomes^57^. Inhibiting v-ATPase with bafilomycin or concanamycin results in inefficient proton pumping into the lumen of endolysosomes and increases in endolysosome pH^58^. The consequences of endolysosome de-acidification are many. Endolysosome de-acidification can inhibit acidic hydrolases, increase non-acid preferring hydrolases, and can lead to the generation of potentially toxic digestion products^46^. Importantly, endolysosome pH dysregulation may contribute to the development of neurodegenerative diseases such as Alzheimer’s disease^46,59^ and HIV-1 associated neurocognitive disorders^60–64^. Therefore, regulators of endolysosome pH may be targeted for identification of novel therapeutics.

Endolysosomes also contain high levels of cations including Ca^2+^, Na^+^, and K^+^ as well as the heavy metals Fe^2+^, Cu^2+^ and Zn^2+^; the release of which is mediated by a variety of endolysosome-resident channels including non-selective TRPML1 cation channels^65,66^. TRPML1 may be also permeable to proton^67,68^. Others and we have found that activating TRPML1 channels with the agonist ML-SA1 resulted in endolysosome acidification and decreasing chloroquine-induced endolysosome de-acidification. These effects of ML-SA1 appear to be specific to TRPML1 because TRPML1 knockdown attenuated the ability of ML-SA1 to decrease endolysosome pH but did not affect basal levels of endolysosome pH. While previous reports have described inconsistent effects of TRPML1 knockdown on endolysosome pH^67–70^, many differences exist between the studies of others and us including cell types, knockdown strategies, and methods for endolysosome pH measurement.

TRPML1 channels can form macromolecular complexes with BK channels and BK channels are activated by TRPML1-mediated calcium release^41^. Because of similarities between functions of TRPML1 and BK channel activation^41,42,71^, we determined whether activation of BK channels acidified endolysosomes. In U87MG cells, not only was there co-localization between BK and TRPML1 channels, but also activating BK channels with NS1619 acidified endolysosomes. Furthermore, we found that BK channel knockdown attenuated the endolysosome acidifying effects of both ML-SA1 and NS1619, whereas TRPML1 knockdown attenuated the endolysosome acidifying effect of ML-SA1 but not that of NS1619. These findings suggest that BK activation following calcium released from TRPML1 channels leads to endolysosome acidification. Although the mechanisms for these effects are not yet clear, increased concentrations of K^+^ in endolysosomes could exchange with protons through actions of a Na^+^(K^+^)/H^+^ exchanger^54,72,73^ or endolysosome membrane hyperpolarization following BK channel activation^41,74^ may enhance the activity or assembly of v-ATPase.

Secreted from HIV-1 infected cells^17–20^, Tat can bind to cell surface receptors including LRP1, heparin sulfate proteoglycan, CD26, and CXCR4 that all participate in receptor-mediated endocytosis^22–25^. Following its endocytosis, Tat enters endolysosomes before escaping into the cytosol, where it can transit to the nucleus and activate the HIV-1 LTR promoter^27–29^. Consistent with others’ findings^22,29,30,32,33^, we demonstrated that chloroquine, a well-known anti-malarial drug that affects morphological features and functions of endolysosomes, increased exogenous Tat-mediated HIV-1 LTR transactivation. A consequence of enhanced HIV-1 LTR transactivation is increased HIV-1 replication and enhanced HIV-1 infectivity. Indeed, not only does chloroquine enhance HIV-1 LTR transactivation, it also enhanced HIV-1 infectivity in cells that require endocytosis for HIV-1 virus entry^75,76^. However, chloroquine did not increase HIV-1 LTR transactivation when Tat was transiently expressed intracellularly^22,35^, under which condition Tat can be imported directly into nucleus and activate HIV-1 LTR transactivation^31^. Thus, chloroquine-induced endolysosome de-acidification likely prevents the degradation of exogenous HIV-1 Tat in endolysosomes and increases the amount of Tat available to activate LTR in the nucleus^22,29,30,32,33^. Furthermore, we found that endolysosome de-acidification by the chloroquine derivative LYS01 and the v-ATPase inhibitor bafilomycin enhanced exogenous Tat-mediated LTR transactivation^34^. Moreover, lipofectamine-based transfection reagents, which avoid lysosome degradation, enhanced exogenous Tat-induced LTR transactivation^77^. Thus, although it remains unclear as to how Tat escapes endolysosomes to enhance LTR transactivation, it is clear that endolysosome pH plays an important role in regulating the cellular levels of Tat and its actions.

Because of our findings that activating TRPML1 with ML-SA1 acidified endolysosome and prevented chloroquine-induced increases in endolysosome pH, we determined whether activating TRPML1 affected Tat-mediated HIV-1 LTR transactivation. ML-SA1 attenuated Tat-mediated HIV-1 LTR transactivation and when TRPML1 was knocked down, ML-SA1-induced restriction in Tat-mediated HIV-1 LTR transactivation was completely prevented. We speculate that endolysosome pH is critical for degradation of Tat degradation via pH-sensitive hydrolysis in endolysosomes. Given that optimum pH is require for efficient enzymatic hydrolysis in endolysosomes, a critical level of endolysosome pH may be required for optimum activities of endolysosomal enzymes that degrade Tat protein efficiently. As such, even though TRPML1 protein was only partially knock-down, ML-SA1 might fail to decrease the endolysosome pH to this critical level, and Tat cannot be degraded efficiently, leaving enough Tat available to mediate HIV-1 LTR transactivation.

**Figure 8:**
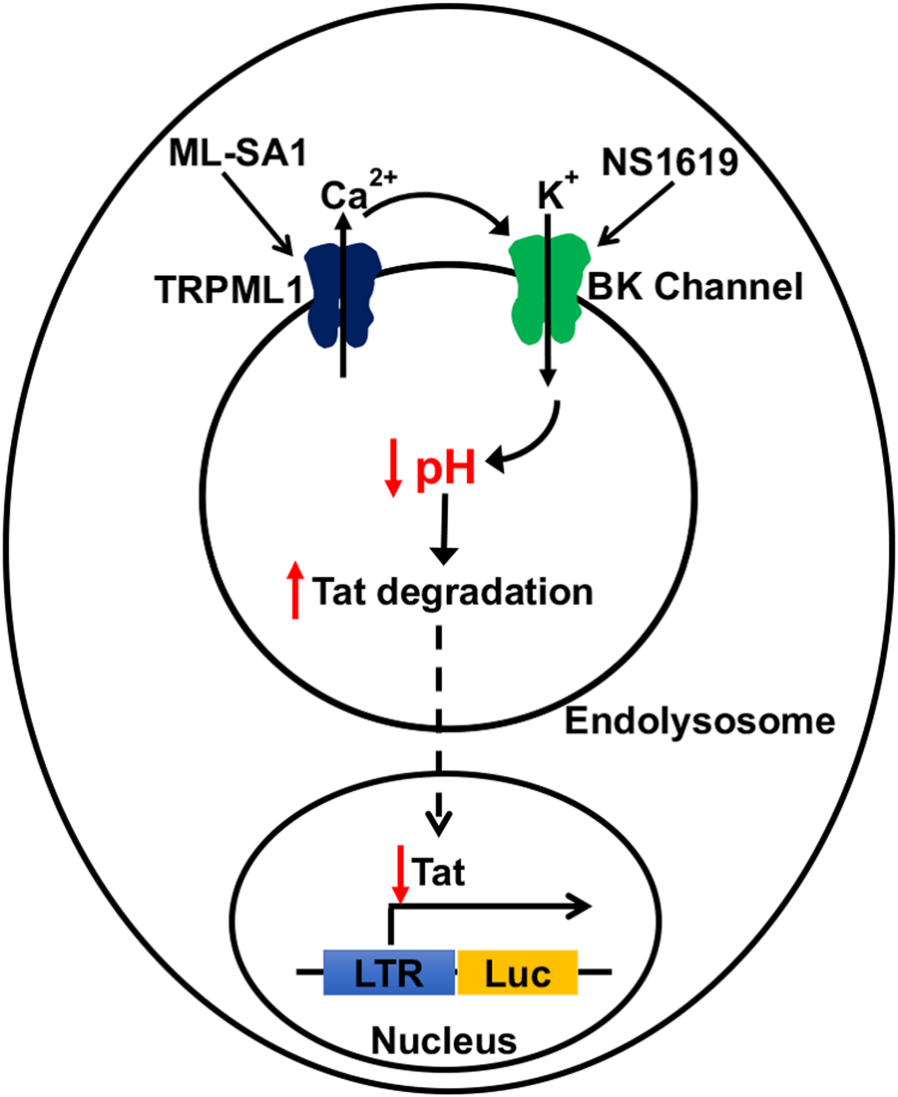
Schematic model for the involvement of TRPML1 and BK channels in the cellular degradation of exogenous Tat. Activating TRPML1 and subsequent activation of BK channels acidifies endolysosomes (decreases endolysosome pH) and enhances degradation of exogenous Tat in endolysosomes, thus reducing the amount of Tat available to activate LTR transactivation in the nucleus.

Because we found that activating BK channels acidified endolysosomes and that pharmacological blocking of BK channels and knocking down BK channels attenuated ML-SA1-induced endolysosome acidification, we next determined the involvement of BK channels in Tat-mediated HIV-1 LTR transactivation. Similar to ML-SA1, activating BK channels restricted Tat-mediated HIV-1 LTR transactivation. In addition, BK channel knockdown prevented the restrictive effect of NS1619 on Tat-mediated HIV-1 LTR transactivation. Furthermore, blocking BK channels with penitrem A and knocking down BK channels prevented ML-SA1-induced restriction in Tat-mediated HIV-1 LTR transactivation, whereas knocking down TRPML1 did not prevent NS1619-induced restriction in Tat-medicated HIV-1 LTR transactivation. In addition, ML-SA1 and NS1619 significantly decreased cellular protein levels of exogenous Tat. Together our data suggests that activating TRPML1 channels and subsequent activation of BK channels acidifies endolysosomes, enhances endolysosome degradation of exogenous Tat, and reduces cellular amount of Tat available to mediate HIV-1 LTR transactivation (Fig. 8).

In summary, our findings indicate that activating TRPML1 channels and subsequent activation of BK channels acidifies endolysosomes, enhances cellular degradation of exogenous Tat, and restricts Tat-mediated LTR transactivation. Our findings suggest that TRPML-1 and BK channels may represent therapeutic targets against HIV-1 infection, HIV-1 associated neurocognitive disorders, and other HIV-1 comorbidities.

## Methods

### Cell culture

U87MG cells were grown in 1X DMEM (Invitrogen) supplemented with 10% fetal calf serum and penicillin/streptomycin (Invitrogen) in a 5% CO_2_ 95% O_2_ incubator at 37 °C temperature. U87MG cells were stably transfected with an integrated luciferase gene under the control of an HIV-1 Tat-driven LTR promoter following neomycin antibiotic (Sigma-Aldrich) selection pressure. Cells were grown to 50% confluence in 35 mm^2^ dishes and 96 well plates, and cells were not used past their tenth passage. These cells were provided generously by Dr. Lena Al-Harthi (Rush University, Chicago).

### Luciferase activity assay for Tat-mediated HIV-1 LTR transactivation

U87MG cells were seeded at a confluency level of about 30–40% (10,000 cells) on 96 well plates one-day prior to being taken for experimentation. Cells were incubated with 2 μg/ml HIV-1 Tat (ABL Inc. and NIH AIDS program) in the presence of 100 μM of chloroquine (Sigma-Aldrich). In some experiments, cells were co-treated with TRPML1 agonist ML-SA1 (Millipore), BK channel blocker Penitrem A (Tocris), or BK channel activator NS1619 (Tocris). 48 hrs post-incubation, luciferase activity assays (Promega) were performed and relative luminescence units were quantified using a fluorometer/luminometer plate-reader (Spectra MAX GEMINI EM).

### Knockdown of TRPML1 and BK channels in U87MG cells

In U87MG cells, expression levels of TRPML1 and BK channels were knocked down using 1 μg/ml shRNA (Santa Cruz) against TRPML1 (Sc-44519) and BK channels (Sc-42511); control shRNA (Sc-108060) was used as a control. Jet prime reagent (2 μl) was used for transfection. Following 36 h incubation, 25 μg/ml puromycin (Invitrogen) was added to activate the shRNA promoter. After incubation for 2 to 3 days, cells were passaged to remove the dead and dying cells, and the remaining living cells were cultured for an additional 36 h prior to being taken for experimentation. Knockdown efficiency was confirmed by immunoblotting using antibodies against TRPML-1 (Abcam, Ab28508) and BK-channel (Abcam, Ab3586).

### Endolysosome pH measurement

Endolysosome pH was measured ratiometrically with LysoSensor (LysoSensor Yellow/Blue DND-160, Invitrogen); a dual excitation dye for pH measurements in acidic organelles^64^. Cells were loaded with LysoSensor (2 µM) for 5 minutes at 37 °C. Light emitted at 510 nm in response to excitation at 340 nm and 380 nm was measured for 20 ms every 5 seconds using a filter-based imaging system (Zeiss) and data were analyzed using Slidebook 6 software (3i). The ratio of light excited at 340/380 nm and emitted at 510 nm was converted to pH using a calibration curve established using 10 µM of the H^+^/Na^+^ ionophore monensin, and 20 µM of the H^+^/K^+^ ionophore nigericin dissolved in 20 mM 2-(N-morpholino) ethane sulfonic acid (MES), 110 mM KCl, and 20 mM NaCl; pH was adjusted between 3.0 to 7.0 with HCl/NaOH.

### Calcium measurement

Free intracellular calcium levels were measured ratiometrically using Fura-2/AM (Invitrogen). The protocol used was basically as described in a previous study of ours^78^. Cells were incubated with 2 µM of Fura-2/AM for 1 hour at 37 °C. After incubation, cells were washed twice with calcium-free media (with 0.2 mM EGTA). Cells were excited at 340 and 380 nm and light emission was captured at 510 nm using our filter-based imaging microscope (Zeiss, Germany). Images were taken using Slidebook 6 (3i) software every 2 seconds; baseline levels and peak increases in free intracellular calcium were determined. The levels of free intracellular calcium levels were represented as a ratio of 340/380 nm.

### Immunoblotting

Cells were harvested and lysed in 1X RIPA lysis buffer (Thermofisher) containing 10 mM NaF, 1 mM Na_3_VO_4_, and protease inhibitor cocktail (Sigma). After centrifugation (14,000 × g for 10 min at 4 °C), supernatants were collected, and protein concentrations were determined with a DC protein assay (Bio-Rad). Proteins (10 μg) were separated by SDS-PAGE (12% gel) and transferred to PVDF membranes accordingly too Iblot2 methods (Invitrogen). The PVDF membranes were incubated overnight at 4 °C with antibodies against GAPDH (Abcam, Ab181603), TRPML1 (Abcam, Ab28508), BK channels (Abcam, Ab3586), and HIV-1 Tat (Santa Cruz, Sc-65916). The blots were developed with enhanced chemiluminescence, and bands were visualized and analyzed by our LI-COR Odyssey Fc Imaging System.

### Immunostaining

Cells were fixed with 1% paraformaldehyde for 10 min followed by cold methanol (−20 °C) for 10 min. The cells were then washed with PBS, blocked with 5% goat serum, and incubated overnight at 4 °C with primary antibodies (1:100) against TRPML1 (Sc-398868, Santa Cruz), BK channels (Ab3586, Abcam) and LAMP1 (D2D11, Cell technology). After washing with PBS, cells were incubated with corresponding Alexa 488- or Alexa 546-conjugated secondary antibodies a(Invitrogen). Cells were examined by confocal microscopy (Zeiss LSM800). Controls for immunostaining specificity included staining neurons with primary antibodies without fluorescence-conjugated secondary antibodies (background controls), and staining neurons with only secondary antibodies; these controls helped eliminate auto-fluorescence in each channel and bleed-through (crossover) between channels.

### Statistical analysis

All experiments were repeated 2–3 times using at least 2 different passages of cells. All data were presented as means and standard deviations. Statistical significance between two groups was analyzed with a Student’s t-test. Statistical significance among multiple groups was analyzed with a one-way ANOVA plus Tukey’s post-hoc test. And, statistical significance among multiple groups involving two factors was analyzed with two-way ANOVA. p < 0.05 was accepted to be statistically significant.

### Ethics approval and consent to participate

No live vertebrates (or higher invertebrates), humans, or human samples were used in any experiments conducted for this manuscript.

## Supplementary information

Supplementary information

## Data Availability

The datasets generated during and/or analyzed during the current study are available from the corresponding author on reasonable request.
