# Supplementary material for: BK channels regulate extracellular Tat-mediated HIV-1 LTR transactivation
Source: Sci Rep. 2019 Aug 22;9:12285. doi: 10.1038/s41598-019-48777-y (PMC6706582; doi:10.1038/s41598-019-48777-y)

BK channels regulate extracellular Tat-mediated HIV-1 LTR transactivation

Nabab Khan, Koffi L. Lakpa, Peter W. Halcrow, Zahra Afghah, Nicole M. Miller,

Jonathan D. Geiger, and Xuesong Chen*

Department of Biomedical Sciences, University of North Dakota School of Medicine and

Health Sciences, Grand Forks, ND 58203, USA

Supplementary Figure S1

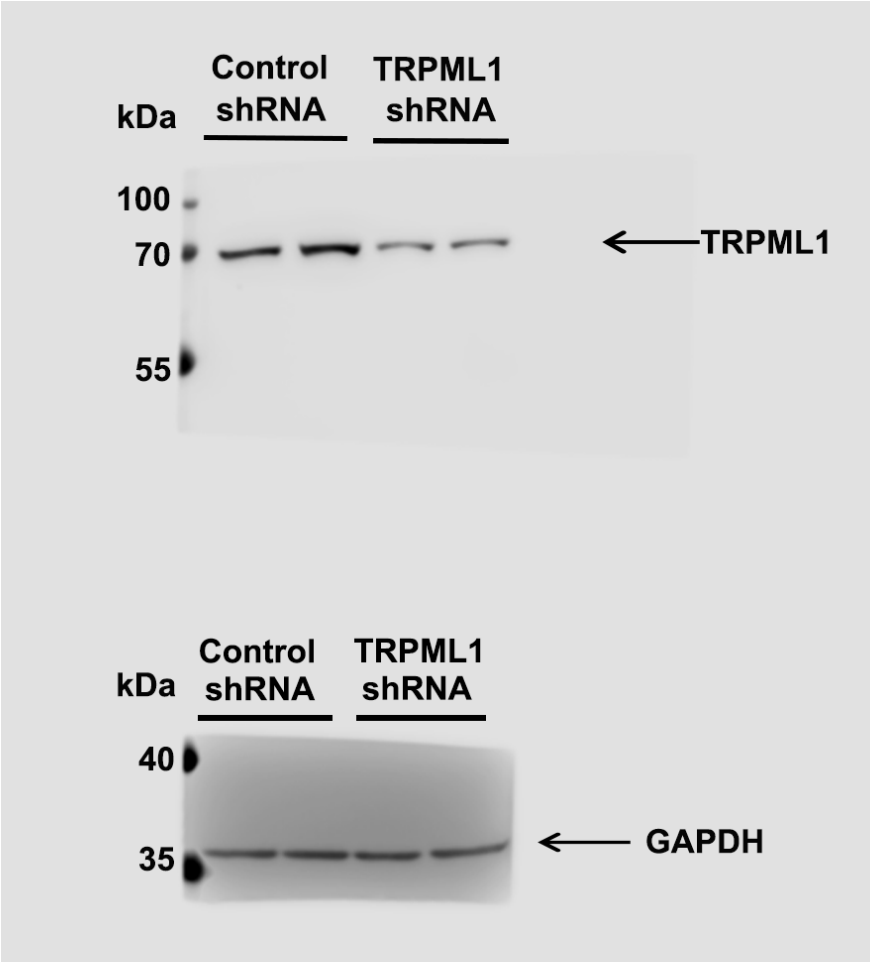

Supplementary Figure S2

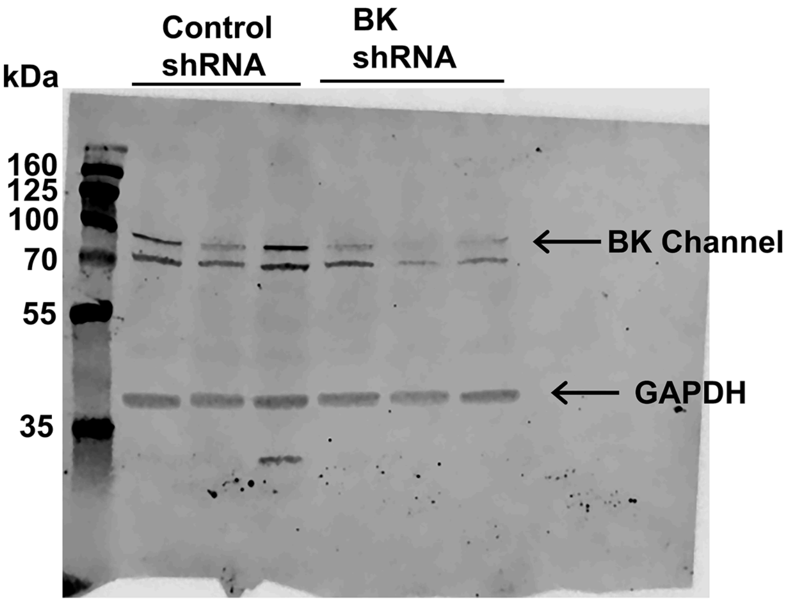

Supplementary Figure S3

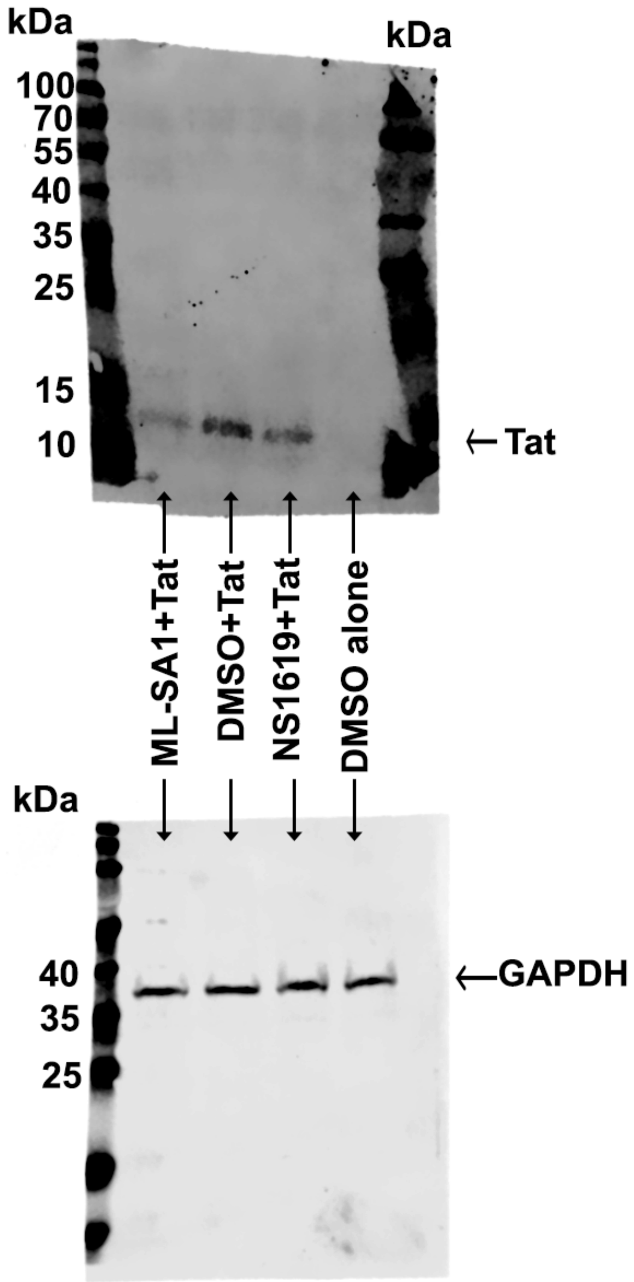

Supplement: Supplementary file 1 — Supplementary information [file 41598_2019_48777_MOESM1_ESM.pdf]
